# Hippocampal neurochemicals are associated with exercise group and intensity, psychological health, and general cognition in older adults

**DOI:** 10.1007/s11357-022-00719-9

**Published:** 2023-01-10

**Authors:** Line S. Reitlo, Jelena M. Mihailovic, Dorthe Stensvold, Ulrik Wisløff, Fahmeed Hyder, Asta Kristine Håberg

**Affiliations:** 1 Department of Neuromedicine and Movement Science, Faculty of Medicine and Health Sciences, Norwegian University of Science and Technology, Trondheim, Norway; 2 Department of Radiology and Biomedical Imaging, Yale University, New Haven, CT USA; 3 Department of Circulation and Medical Imaging, Faculty of Medicine and Health Sciences, Norwegian University of Science and Technology, Trondheim, Norway; 4 School of Human Movement and Nutrition Science, University of Queensland, Brisbane, Australia; 5 Department of Radiology and Nuclear Medicine, St. Olavs Hospital, Trondheim University Hospital, Trondheim, Norway

**Keywords:** Cardiorespiratory fitness, Neuroimaging, Intervention, Elderly, Limbic

## Abstract

Based on the premise that physical activity/exercise impacts hippocampal structure and function, we investigated if hippocampal metabolites for neuronal viability and cell membrane density (i.e., N-acetyl aspartate (NAA), choline (Cho), creatine (Cr)) were higher in older adults performing supervised exercise compared to following national physical activity guidelines. Sixty-three participants (75.3 ± 1.9 years after 3 years of intervention) recruited from the Generation 100 study (NCT01666340_date:08.16.2012) were randomized into a supervised exercise group (SEG) performing twice weekly moderate- to high-intensity training, and a control group (CG) following national physical activity guidelines of ≥ 30-min moderate physical activity ≥ 5 days/week. Hippocampal body and head volumes and NAA, Cho, and Cr levels were acquired at 3T with magnetic resonance imaging and spectroscopic imaging. Sociodemographic data, peak oxygen uptake (VO_2peak_), exercise characteristics, psychological health, and cognition were recorded. General linear models were used to assess group differences and associations corrected for age, sex, education, and hippocampal volume. Both groups adhered to their training, where SEG trained at higher intensity. SEG had significantly lower NAA/Cr in hippocampal body than CG (*p* = 0.04). Across participants, higher training intensity was associated with lower Cho/Cr in hippocampal body (*p* < 0.001). Change in VO_2peak_, increasing VO_2peak_ from baseline to 3 years, or VO_2peak_ at 3 years were not associated with hippocampal neurochemicals. Lower NAA/Cr in hippocampal body was associated with poorer psychological health and slightly higher cognitive scores. Thus, following the national physical activity guidelines and not training at the highest intensity level were associated with the best neurochemical profile in the hippocampus at 3 years.

## Introduction

Physical activity and/or exercising are suggested as avenues to preserve brain function and structure in older adults and even to stave off dementia [1–4]. The hippocampus is considered a brain region particularly adaptable to the positive effects of physical activity and/or exercising, while also being highly susceptible to pathology associated with Alzheimer’s disease and vascular dementia [5–8]. In animal studies, exercise is demonstrated to increase hippocampal neurogenesis and improve memory functions, supporting the notion that exercising is particularly relevant for preserving hippocampal structure and function [9–13]. In humans, structural magnetic resonance image (MRI) studies have demonstrated larger hippocampal volumes, perhaps located to the hippocampal head, in more physically active or exercising individuals in intervention and randomized control trials [7, 14–17]. However, either no effects or even negative effects of exercising on the hippocampus volume have also been described [18–24]. Likewise, inconsistent results have also been reported between physical activity or exercising and cognition in humans [25–28].

Investigations into the molecular characteristics of the hippocampus might provide new insight into mechanisms through which physical activity and/or exercise impact both brain structure and function. While structural MRI of the hippocampus is widespread and well known, the measurement of cerebral metabolite levels *in vivo* by magnetic resonance spectroscopic imaging (MRSI) is less frequently applied. Metabolites commonly identified include N-acetyl aspartate (NAA) and choline-containing compounds, often referred as choline (Cho) and creatine (Cr). NAA is used as a proxy for neuronal viability with diminished levels of NAA found in conditions such as Alzheimer’s disease [29], mild cognitive impairment [30], and normal aging [31], suggesting neuronal or synaptic losses in these conditions. Cho-containing molecules are components of membrane phospholipids, with decreased or increased levels suggesting changes in number of membranes related to for instance cellular density, turnover, breakdown, or inflammation, and associated with cognitive impairments in aging [32]. Cr provides a proxy measure of cellular energy and represents the sum of creatine and phosphocreatine which are present in both neurons and glial cells. This signal is often used as an internal standard for comparison of metabolites across different groups/conditions [33].

NAA/Cr or Cho/Cr levels are hence considered biomarkers of neuronal health and neural cell membrane density, respectively. Even though MRSI is available on clinical scanners, this technique is seldom employed compared to structural MRI. MRSI in the hippocampus, a core region of interest in the study of the effects of exercise and physical activity on the brain, has not been performed. So far, only one observational study has investigated the effect of exercising on brain metabolite levels in cortex using single-voxel magnetic resonance spectroscopy (MRS) [34]. They reported that middle-aged adults (45–60 years) who performed endurance training on a regular basis had higher NAA/Cr and Cho/Cr levels in frontal and occipitoparietal cortices, respectively, compared to their sedentary counterparts. Furthermore, maximal oxygen uptake (VO_2max_) was positively associated with frontal NAA/Cr and occipital Cho/Cr levels.

The aim of this study was to assess NAA/Cr and Cho/Cr in the hippocampal head and body in older adults taking part in the Generation 100 study, a randomized clinical trial (RCT) of supervised exercise versus following national physical activity guidelines [35]. Previous studies suggest a particularly beneficial effect of exercising on the hippocampal head [14] while atrophy of CA1 subfield of the hippocampal body is associated with risk of Alzheimer’s disease [36]. We therefore employed MRSI with a semi-localized adiabatic selective refocusing (sLASER) pulse sequence [37] to acquire metabolic profiles from voxels in the hippocampal head and body. Hippocampal head and body volumes from structural MRI were used to correct for volume differences. We predicted that the supervised exercise group (SEG) would have a higher NAA/Cr and Cho/Cr in the hippocampal head compared to the control group (CG) after 3 years of intervention. In line with the cardiorespiratory fitness hypothesis [38], we assumed that NAA/Cr and Cho/Cr levels in both hippocampal head and body would be positively associated with gains in peak oxygen uptake (VO_2peak_) as well as VO_2peak_ at time of MRSI. We explored associations between measures related to exercising and physical activity, i.e., its intensity and duration, to evaluate possible association between these and hippocampal metabolites to extend the knowledge on how exercising may influence the brain. Finally, we investigated if NAA/Cr and Cho/Cr were associated with psychological distress and general cognition at 3 years to link our results to clinical outcomes.

## Methods

### Ethics

The RCT and the substudy were approved by the Regional Committee for Medical Research Ethics (REK 2012/381 B and REK 2012/849, respectively). Both studies adhered to the Helsinki Declaration and participants gave their written informed consent to both.

### The RCT study

Participants were from the RCT Generation 100 study (NCT01666340, ClinicalTrials.gov registry) which originally set out to evaluate the effect of 3 years of supervised exercise training versus following the national physical activity guidelines on morbidity and mortality in older adults. The MRS acquisition was planned to be an outcome after end of the intervention at 3 years. At 3 years, the study period was extended to 5 years due to lower mortality in the cohort than anticipated based on national numbers [35]. MRS was, however, not repeated at the new endpoint.

All inhabitants of Trondheim, Norway, born between 1936 and 1942 registered in the National Population Registry (*n* = 6966) received an invitation letter in 2012. A total of 1790 declared interest while 1422 declined. Consequently, 1567 persons (790 women) were invited to baseline examination; 174 did not show up or withdrew and 49 were excluded. The inclusion criterion was having the physical ability to take part in an exercise intervention. Exclusion criteria were somatic diseases precluding vigorous physical activity or associated with a significantly shortened life expectancy, dementia, and any condition which limited ability to exercise as well as participating in another exercise study.

Participants were stratified by sex and cohabitation status before being randomized into a supervised exercise group performing either high-intensity interval training (HIIT) (*n* = 400) or moderate-intensity continuous training (MICT) (*n* = 387), and a control group (*n* = 780). Before randomization in the Generation 100 study, the participants were asked if they were interested in also taking part in a brain MRI study. Baseline data collection before the start of the intervention was performed between August 2012 and June 2013.

### The Generation 100 MRI study

**Fig. 1:**
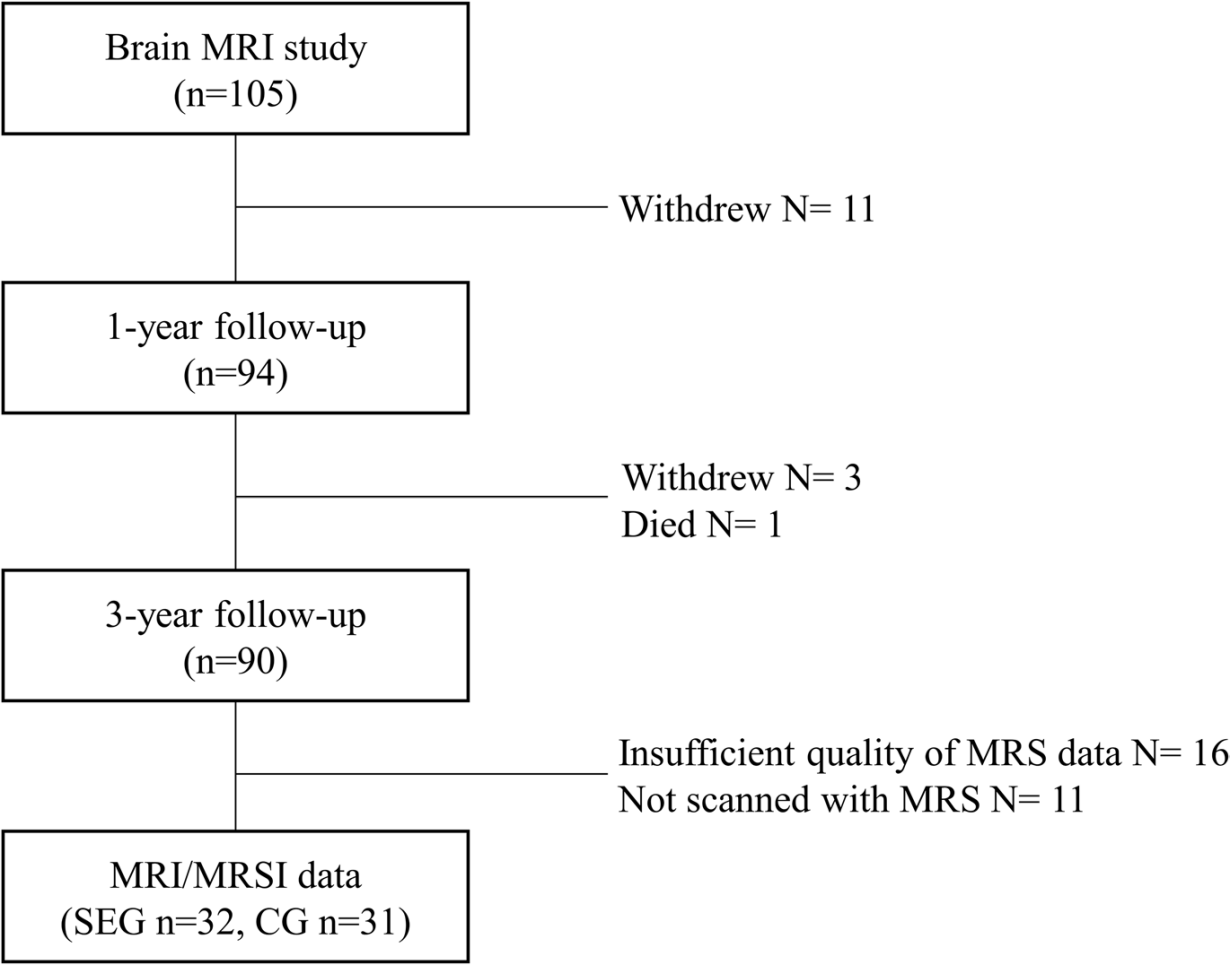
Flowchart describing inclusion of participants. Number (*n*) of participants and reason for exclusion (withdrew, died, insufficient quality, or lack of data) are presented

A total of 111 (55 men, 56 women) agreed to participate. Six were excluded due to MRI contraindications or previous neurosurgical disorders, leaving 105 participants (53 men, 52 women). The participants underwent a standardized structural MRI protocol [20], and at the 3-year follow-up, MRSI data was acquired. In total, 90 participants were still in the MRI study at the 3-year follow-up, of which 11 participants were not scanned with MRSI due to inability to stay in the scanner after the structural scans were acquired. MRSI data from another 16 participants could not be used due to insufficient quality because of movement (Fig. 1).

### Interventions

Participants in the SEG were randomized to twice weekly MICT or HIIT sessions. The MICT sessions consisted of 50 min of continuous exercise at about 70% of peak heart rate corresponding to a rating of perceived exertion of about 13 on the Borg scale [39]. The HIIT sessions started with 10-min warm-up followed by 4 × 4 min intervals at about 90% peak heart rate corresponding to a rating of perceived exertion of about 16 [40]. Every 6^t^^h^ week, participants in the SEG attended mandatory spinning classes where they exercised with a heart rate monitor to ensure compliance with the prescribed training intensity. The participants in the active control group were asked to follow the Norwegian physical activity guideline for 2012, which states at least 30 min of moderate-level physical activity at least 5 days a week [35]. Yearly questionnaires were used to assess adherence to the allocated program, defined as performing at least 50% of the prescribed exercise sessions or physical activity recommendations (for details, see [40]).

### Sociodemographic, health, fitness, and exercise variables

Date of birth, sex, and level of education (primary school, high school, and university) were obtained at baseline [35]. Data on cohabitation status and current smoking (yes/no) was obtained from annual questionnaires. Psychological health was assessed with a Norwegian validated version of the HADS questionnaire [41, 42]. Total HADS score was used [43, 44]. The Norwegian validated version of Montréal Cognitive Assessment (MoCA) scale was administrated [45] and total score reported.

Clinical measurements, including body mass index (BMI), blood pressure, and resting heart rate, were acquired at baseline and after 3 years of intervention using standard practices [35]. At the same timepoints, fasting blood samples were obtained, and high-density lipoprotein (HDL), low-density lipoprotein (LDL), triglycerides (TG), blood glucose, hemoglobin A1c (HbA1C), and high-sensitivity C-reactive protein (hsCRP) were assessed.

Cardiorespiratory fitness was assessed objectively as VO_2peak_ using graded maximal exercise testing on a treadmill or exercise bike at baseline and 3-year follow-up [46]. Participants with previous heart diseases were tested under ECG monitoring, and participants with known cardiovascular disease were tested according to the American College of Cardiology/American Heart Association guidelines for exercise testing of patients with known cardiovascular disease [47]. Maximal oxygen uptake (VO_2max_) was reached when the respiratory exchange ratio was ≥ 1.05 and the participant did not increase oxygen uptake more than 2 mL during the last 30 s despite the increased workload. If these criteria were not met, participants were described as having reached VO_2peak_. Many participants (see Table 2) did not meet the criteria for VO_2max_ which is common in aging populations [40], and instead reached VO_2peak_. Only VO_2peak_ data from participants who stopped the test due to exhaustion were included while those who stopped due to factors such as pain or lack of motivation were excluded. VO_2max_ and VO_2peak_ data were combined depending on which was achieved by each participant, into a variable termed VO_2peak_.

A physical activity questionnaire including questions on frequency and duration of exercise was used to calculate weekly exercise duration (min/WK) at 3 years (for details, see [40]), while the Borg 6–20 rating of perceived exertion scale [39] was used to assess exercise intensity.

### Brain MRI and MRSI

**Fig. 2:**
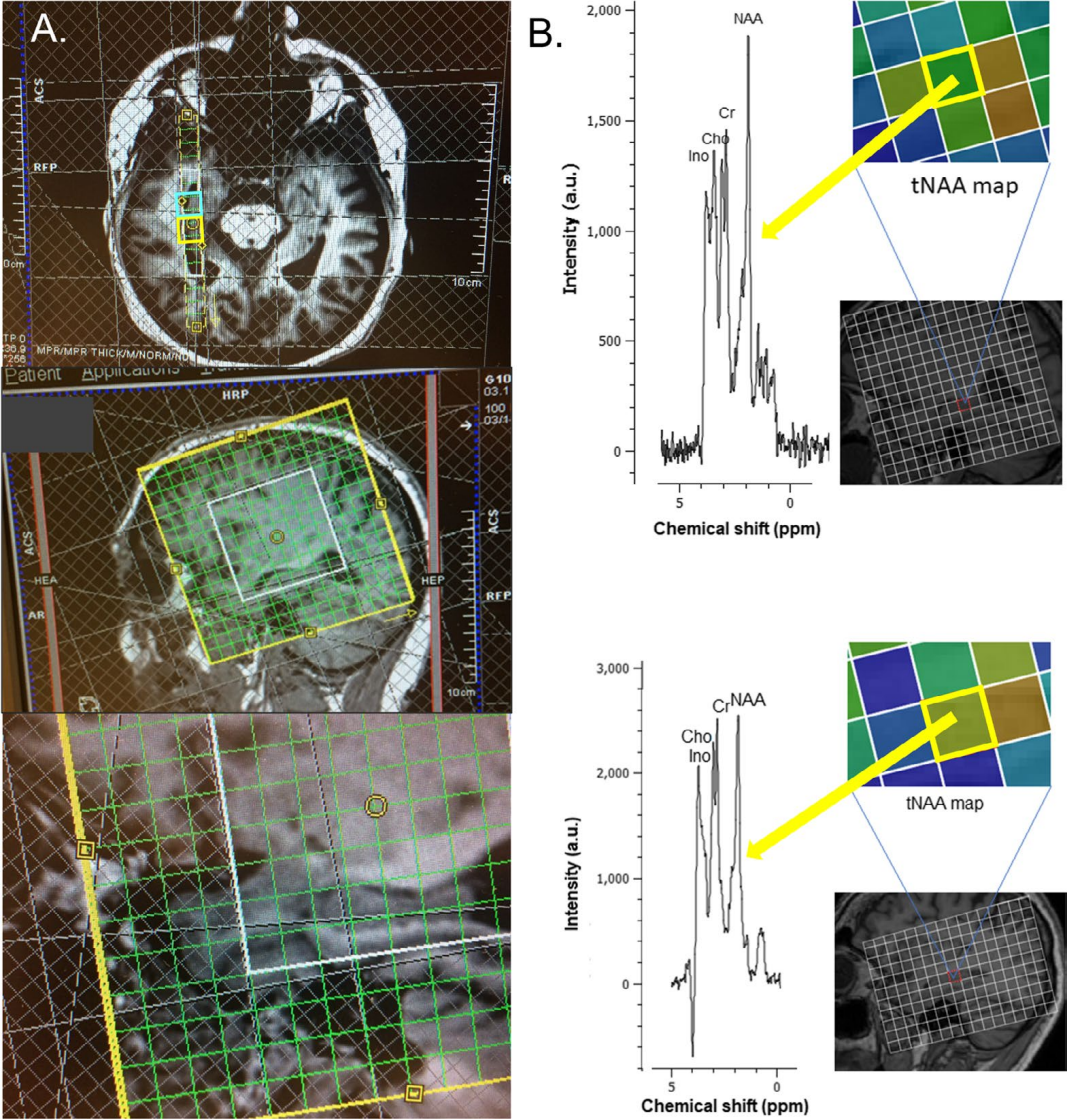
Magnetic resonance spectroscopic imaging (MRSI) acquisition and representative spectra. **A** Positioning of the MRSI volume/grid in one Generation 100 study participant. The grid was angulated along the hippocampal long axis. The tip of the hippocampal head was always the third lower row voxel within the white outlined 16 × 16 grid. In the upper image, the turquoise square outlines the hippocampal head voxel, while the yellow square outlines the hippocampal head voxel. **B** Typical spectra from the hippocampal body. The upper spectrum is from a participant in CG and the lower spectrum from a participant in SEG. Metabolites shown in spectra (left) are N-acetyl aspartate (NAA), creatine (Cr), choline (Cho), and myoinositol (Ino). The MRSI maps (right) reflect the total NAA (tNAA) map for respective subjects

MRI/MRSI data were acquired on 3T Magnetom Skyra scanner (Siemens AG, Erlangen, Germany) equipped with a 32-channel head coil for homogenous signal-to-noise ratio (SNR) due to radio frequency (RF or B_1_) uniformity. Following advanced static magnetic field (B0) shimming, the MRI scan used in this study, a high-resolution 3D T1-weighted MPRAGE (TR = 1900; TE = 3.16; FOV = 256 × 256; slice thickness = 1 mm; gap = 0 mm), was acquired. From the T1-weighted MPRAGE scans, we obtained hippocampal long-axis volumes, i.e., hippocampal head, body, and tail volumes, in Freesurfer v. 6.0 (http://surfer.nmr.mgh.harvard.edu/) [48]. The MPRAGE scan was used for positioning of the MRSI grid which was angulated parallel to right hippocampus long axis (Fig. 2). A 2D MRSI method based on sLASER sequence with 4 preparation scans in a 16 × 6 × 1 grid with TR = 1700 ms, TE = 40 ms, flip angle = 90°, water suppression band width = 50 Hz, averages = 6, field of view = 120 × 120, volume = 60 × 60 mm, and slice thickness 10 mm was used.

### MRSI processing and metabolite quantification of NAA, Cho, and Cr

MRSI processing consisted of two main steps: preprocessing and fitting. Preprocessing included zero filling, water peak removal, phase correction, baseline correction, eddy current, and noise filtering. Although water suppression was used during MRSI acquisition, the spectra still contained a residual water peak. Usually, this peak width is in the frequency range < 45 Hz. We used Hankel singular value decomposition (HSVD) filter [49] where the water signal is first estimated using a subspace-based decomposition into a sum of complex damped exponentials and subsequently removed from the measured signal to suppress the water component in the specified frequency region, while the other frequency regions are minimally affected. For baseline correction, local minima of spectra were found and then iteratively generated envelopes were averaged and used to estimate baseline, which was subtracted from spectrum to generate baseline-corrected spectrum.

Metabolite quantification was achieved with LCModel version 6.3-1K (Stephen Provencher, Inc., Oakville, Ontario, Canada) [50]. Chemical shift and J-coupling values from Govindaraju et al. were used to simulate the metabolites, which included lipids and macromolecules to get the basis set [51]. Each metabolite was simulated with a Lorentzian lineshape with a full width at half maximum of 0.8 Hz. The accuracy of this basis set compared with experimentally derived metabolite spectra has been previously demonstrated [52]. Simulation of the collection of basis functions (basis set) is the preferred method in regions such as the hippocampus where MR spectra can be of lesser quality due to for instance partial volume effects [53–55]. However, the first few points of the FID contain very broad signals, not easily modeled with a basis set. Therefore, starting the fitting point was set to be 24th data point with endpoint set to 2048.

^1^H-MRSI techniques use rectangular or cubic voxels, which do not usually correspond with the curved shapes of the hippocampus. Hence, MRSI voxels can include a combination of cerebrospinal fluid (CSF), gray matter (GM), and white matter (WM). Because CSF has no measurable metabolites, the presence of a large portion of CSF within the voxel could underestimate the metabolite concentrations. Furthermore, metabolite concentrations are different in GM and WM portions [56, 57]. When a voxel contains a heterogeneous tissue composition, the spectroscopic signal acquired from this voxel will consist of the signal from different tissues making reliable measurements difficult. To avoid contribution of different tissues and CSF inside voxels, we did partial volume correction [58–61]. LCModel quantification accuracy was assessed through Cramer-Rao lower bounds (CRLB) [62]. Metabolites quantified with a CRLB above 50% were considered not detected. The average CRLB for NAA, Cho, and Cr were 6.9%, 7.0%, and 5.5%, respectively. The LCModel fitting was performed over the spectral range from 0 to 5 ppm. Total NAA, Cho, and Cr values were quantified and reported as NAA/Cr and Cho/Cr ratios.

### Statistics

Mean, standard deviation (SD), 95% confidence intervals (CI), and % were used as appropriate to display the distribution of variables. Statistical comparisons of sociodemographic, health, fitness, and exercise variables were performed with *t*-tests, Mann–Whitney *U*, and Pearson chi-square as appropriate. Effect size calculated using Cohen’s *d* was provided for NAA/Cr and Cho/Cr in the hippocampal head and body. Across all subjects, a paired *t*-test was used to compare differences in the spatial distribution of NAA/Cr and Cho/Cr between voxels in the hippocampal head and body.

Group differences in NAA/Cr and Cho/Cr in the hippocampal head and body were assessed in general linear models including the covariates sex, age, education, and volume of the hippocampal head or body, respectively.

Since increasing VO_2peak_ is suggested to mediate the positive effects of exercise on the brain, we investigated if degree of change in VO_2peak_ (VO_2peak 3-years_ - VO_2 peak Baseline_) was associated with NAA/Cr and Cho/Cr in the hippocampal head and body in general linear models. Subsequently, we stratified participants into groups with increasing (54.5%) versus decreasing (45.5%) VO_2peak_ from baseline to 3 years and evaluated the presence of a group difference. Finally, we investigated the association between VO_2peak_ at 3 years with NAA/Cr and Cho/Cr. These analyses were performed across all participants (SEG&CG) in general linear models including sex, age, education, and volume of the hippocampal head or body as covariates. Sensitivity analyses were performed limited to those reaching VO_2max_.

To investigate potential associations between variables related to exercising at time of MRS, we assessed the relationships between NAA/Cr and Cho/Cr and weekly exercise duration and intensity across all subjects in general linear models including sex, age, education, and volume of the hippocampal head or body as covariates.

Finally, we investigated if HADS and MoCa scores were associated with NAA/Cr or Cho/Cr in the body or head of the hippocampus across all subjects (SEG&CG) using one general linear model for NAA/Cr and one for Cho/Cr with sex, age, education, and hippocampal volume at 3 years, to evaluate the potential clinical impact of these MRSI biomarkers.

Based on the group sizes in the only previously published exercise and brain metabolite study, *n* = 28 in each group was considered a sufficient number of participants needed to detect a group difference [34].

SPSS (IBM Corp. Released 2010. IBM SPSS Statistics for Windows, Version 28.0. Armonk, NY: IBM Corp) was used for all analyses. The statistical significance threshold was set to *p* < 0.05.

## Results

Usable spectral data were obtained in 63 (32 SEG/31 CG) of the 79 participants in whom MRSI scans were conducted. Motion, small hippocampal volumes, and technical processing/analysis issues were reasons for lack and exclusion of MRSI data. There were no significant differences in sociodemographic variables or VO_2peak_ at 3 years between participants with and without MRSI data (Table 1).

**Table 1:** Sociodemographic variables and VO_2peak_ for participants in the Generation 100 brain study with (MRSI +) and without (MRSI −) MRSI data at 3 years

	G100 MRI cohort		*p*
	MRSI −	MRSI +	
Number of participants^a^ (M/W)	13/14	32/31	0.82
Age^b^ (years)	75.9 (1.8)	75.3 (1.9)	0.20
Education^c^			0.39
%Primary school	18.5	14.5	
%High school	18.5	12.9	
%University	63.0	72.6	
VO_2peak_ (mL/kg/min)^b^	29.6 (7.0)	31.7 (7.5)	0.25

Continuous measures are presented as mean with standard deviation in parentheses. Categorical data is reported as percentages. Significance threshold was set to *p* < 0.05, ^a^Pearson chi-square, ^b^*t*-test, ^c^Mann-Whitney *U* test. Abbreviations: *MRSI* + , successful MRSI acquisition and analysis; *MRSI − *, unusable MRSI data; *M*, men; *W*, women

### Sociodemographic, health and fitness, and exercise variables

An overview of the sociodemographic and health characteristics of the included participants in the two groups at baseline and after 3 years is presented in Table 2. There were a similar number of women and men in both groups, and no significant differences between the groups in any of the variables at baseline or 3-year follow-up. VO_2peak_ was not significantly different in the two groups at baseline or after 3 years.

**Table 2:** Sociodemographic and clinical variables for the supervised exercise (SEG) and control groups (CG) at inclusion and after 3 years of intervention

	Baseline			3-year follow-up		
	CG	SEG	*p*	CG	SEG	*p*
Number of participants^a^ (M/W)	15/16	17/15	0.71	15/16	17/15	0.71
Age^b^ (years)	71.6 (1.7)	71.9 (2.0)	0.54	75.1 (1.8)	75.4 (2.1)	0.62
Education^c^			0.94			0.94
%Primary school	3.2	9.4		3.2	9.4	
%High school	25.8	18.8		25.8	18.8	
%University	71.0	71.9		71.0	71.9	
Living alone^a^ (%yes)	29.0	25.8	0.78	31.0	26.7	0.71
Current smoker^a^ (% no)	96.8	93.3	0.54	93.1	93.3	0.97
BMI^b^ (kg/m^2^)	25.7 (3.4)	26.2 (3.5)	0.53	25.9 (3.2)	26.2 (3.5)	0.31
RHR^b^ (beats/min)	66.9 (8.9)	66.4 (9.9)	0.85	61.6 (9.0)	62.0 (8.9)	0.89
DBP^b^ right (mmHg)	74.3 (8.8)	76.4 (8.3)	0.34	72.5 (8.2)	74.4 (9.3)	0.42
SBP^b^ right (mmHg)	132.0 (16.5)	132.5 (17.0)	0.91	132.8 (14.9)	130.0 (18.2)	0.53
HDL^b^ (mmol/L)	2.0 (0.6)	1.8 (0.5)	0.15	2.1 (0.6)	1.8 (0.5)	0.09
LDL^b^ (mmol/L)	3.6 (1.0)	3.3 (0.8)	0.33	3.7 (1.0)	3.2 (0.8)	0.06
TG^b^ (mmol/L)	1.0 (0.4)	1.0 (0.5)	0.61	0.8 (0.07)	1.0 (0.1)	0.10
Glucose^b^ (mmol/L)	5.6 (0.6)	5.4 (5.5)	0.30	5.4 (0.5)	5.4 (0.5)	0.72
HbA1c^c^ (%)	5.5 (0.2)	5.6 (0.4)	0.63	5.4 (0.04)	5.5 (0.5)	0.42
hsCRP^b^ (mg/L)	1.4 (0.9)	2.9 (5.4)	0.93	4.1 (11.2)	1.8 (2.2)	0.40
HADS^b^ (total score)	4.8 (3.8)	4.2 (3.9)	0.51	5.5 (4.5)	4.9 (4.3)	0.59
MoCA^b^ (mean score)				27.0 (2.2)	27.2 (2.1)	0.83
VO_2peak_ (mL/kg/min)^b^	31.3 (6.4)	30.5 (6.9)	0.62	31.7 (8.2)	31.7 (6.9)	0.99
VO_2peak_ or VO_2max_^a#^ (%VO_2max_)	64.5	65.6	0.93	76.9	58.6	0.15

The continuous measures are shown as mean and standard deviation in the parentheses. Categorical data is reported as percentages. Significant threshold was set to *p* < 0.05. ^a^Pearson chi-square, ^b^*t*-test, ^c^Mann-Whitney *U* test; ^d^MoCA was not performed at baseline. ^#^Percentage of participants that achieved VO_2max_ during maximal exercise testing. Abbreviations: *CG*, control group; *SEG*, supervised exercise group; *M*, men; *W*, women; *BMI*, body mass index; *RHR*, resting heart rate; *DBP*, diastolic blood pressure; *SBP*, systolic blood pressure; *HDL*, high-density lipoprotein; *LDL*, low-density lipoprotein; *HbA1c*, glycated hemoglobin; *hsCRP*, high-sensitivity C-reactive protein; *TG*, triglycerides; *HADS*, Hospital Anxiety and Depression Scale; *MoCA*, Montréal Cognitive Assessment Scale; *VO*_*2peak*_, peak oxygen uptake

Both CG and SEG adhered similarly well to their respective regimes, with an adherence of 87.5% and 84.4%, respectively, at 3 years (Table 3). There was no significant difference in average weekly exercise duration between the groups, but exercise intensity and the percentage of participants exercising per the HIIT protocol were significantly higher in SEG than CG (Table 3).

**Table 3:** Adherence, exercise intensity, and duration for the supervised exercise (SEG) and control group (CG) at 3 years

	CG	SEG	*p*
Adherence^a^	87.1	84.4	0.76
Exercised per HIIT protocol^a#^	27.6	55.2	**0.03**
Exercise intensity^b^	13.5 (2.2)	14.7 (1.7)	**0.02**
Exercise duration (min/week)^b^	145.1 (83.0)	154.8 (66.7)	0.63

Adherence and exercised per HIIT protocol are reported in percentages, while exercise intensity and duration as mean with standard deviation in parenthesis. *p*-values < 0.05 are in bold font. ^a^Pearson chi-square, ^b^*t*-test. Abbreviations: *CG*, control group; *SEG*, supervised exercise group; *HIIT*, high-intensity interval training. ^#^Percentage of participants exercising per the HIIT group protocol defined as exercising at least ≥ 30 min at ≥ 15 on the Borg scale per week (for details, see [40])

### Hippocampal metabolite ratios, intervention groups, VO_2peak_, and exercise variables

The observed mean, SD, 95% CI, and Cohen’s *d* effect size of NAA/Cr and Cho/Cr ratios in the voxels in the hippocampal head and body for each group are presented in Table 4. For NAA/Cr ratios in the hippocampal body and head, moderate differences were present, while for Cho/Cr, small to moderate effect sizes were found between SEG and CG. The paired comparison of NAA/Cr and Cho/Cr levels, respectively, showed higher NAA/Cr levels in the hippocampal body than head within the subjects.

**Table 4:** Mean, standard deviation (SD), and 95% confidence interval for the raw observations of NAA/Cr and Cho/Cr ratios in the hippocampal body and head in the control group (CG) and supervised exercise group (SEG)

		n_CG/SEG_	CG			SEG			Effect size#	All (CG&SEG)			p*
			Mean	SD	95% CI	Mean	SD	95% CI		Mean	SD	95% CI	
NAA/Cr	Body	21/25	1.06	0.22	[0.97–1.15]	0.87	0.32	[0.74–1.00]	0.28	0.95	0.29	[0.87–1.04]	0.012
	Head	24/16	0.89	0.27	[0.78–1.00]	0.83	0.39	[0.63–1.02]	0.32	0.87	0.32	[0.77–0.97]	
CHO/Cr	Body	22/25	0.36	0.11	[0.31–0.41]	0.33	0.11	[0.29–0.37]	0.11	0.32	0.22	[0.26–0.38]	0.83
	Head	23/17	0.38	0.30	[0.26–0.50]	0.26	0.09	[0.22–0.30]	0.22	0.35	0.11	[0.32–0.38]	

CG*,* control group; *SEG*, supervised exercise group; *N*_*CG/SEG*_, number of participants in CG and SEG; *SD*, standard deviation; *CI*, confidence interval. #Calculated with Cohen’s *d*. *p-values from paired *t*-test

The general linear model revealed a significant effect of group on NAA/Cr ratio in the hippocampal body with lower levels in SEG compared to CG after controlling for age, sex, education, and hippocampal body volume. No significant group effect was uncovered for Cho/Cr in hippocampal body or for NAA/Cr or Cho/Cr in the hippocampal head (Table 5).

**Table 5:** Effect of intervention group on and associations of exercise measures with NAA/Cr and Cho/Cr levels in the hippocampal body and head as outcome variables

	NNA/Cr						Cho/Cr					
	Body			Head			Body			Head		
	B	CI	*p*	B	CI	*p*	B	CI	*p*	B	CI	*p*
Intervention group^#^	0.19	[0.01–0.37]	**0.04**	0.03	[− 1.04–7.79]	0.82	0.11	[− 0.04–0.26]	0.14	0.03	[− 0.15–2.84]	0.42
VO2_peak_ 3 years–baseline^$^	− 0.03	[− 0.06–0.01]	0.09	0.00	[− 0.03–0.03]	0.98	0.01	[− 0.02–0.03]	0.61	0.01	[− 0.01–0.01]	0.63
VO_2peak_ group^#^	− 0.05	[− 0.28–0.19]	0.69	0.10	[− 0.20–0.40]	0.51	0.11	[− 0.08–0.29]	0.25	0.03	[− 0.05–0.12]	0.43
VO_2peak_ 3 years^$^	0.01	[− 0.02–0.02]	0.75	− 0.01	[− 1.17–8.68]	0.34	0.01	[− 0.01–0.02]	0.88	0.01	[− 0.01–0.01]	0.62
Exercise duration/week, 3 years^$^	0.01	[0.00–0.01]	0.14	0.00	[− 0.01–0.01]	0.56	− 0.01	[− 0.01–0.00]	0.06	0.00	[− 0.01–0.00]	0.56
Exercise intensity, 3 years^$^	− 0.01	[− 0.07–0.05]	0.80	− 0.01	[− 0.06–0.05]	0.92	− 0.06	[− 0.09– − 0.03]	**0.001**	− 0.01	[− 0.03–0.10]	0.35

#Group effect, control group > supervised exercise group, decreasing VO_2peak_ versus increasing Vo_2peak_, ^$^Association across all participants (SEG&CG). A *p*-value < 0.05 was considered significant and marked in bold font. General linear models with NAA/Cr or Cho/Cr body/head as outcome variables corrected for sex, age, education, and hippocampal head/body volume

There were no associations between change in VO_2peak_ from baseline to 3 years across all participants (SEG&CG) and hippocampal metabolites correcting for age, sex, education, and hippocampal head or body volume (Table 5). There was also no difference in hippocampal metabolite ratios between the participants in the increasing VO_2peak_ versus the declining VO_2peak_ group (Table 5). Furthermore, there were no significant associations between VO_2peak_ at 3 years and hippocampal metabolites at 3 years. Sensitivity analyses with only participants reaching VO_2max_ (*n* = 37) revealed the same results (results not shown).

Across all participants (SEG&CG), weekly exercise duration was not associated with NAA/Cr or Cho/Cr in neither the hippocampal head nor body, correcting for age, sex, education, and hippocampal head or body volume. Higher exercise intensity at 3 years was associated with lower Cho/Cr level in the hippocampal body correcting for age, sex, education, and hippocampal body volume. No other associations were present for exercise intensity (Table 5).

### Hippocampal metabolite ratios and associations with HADS and MoCA scores

**Fig. 3:**
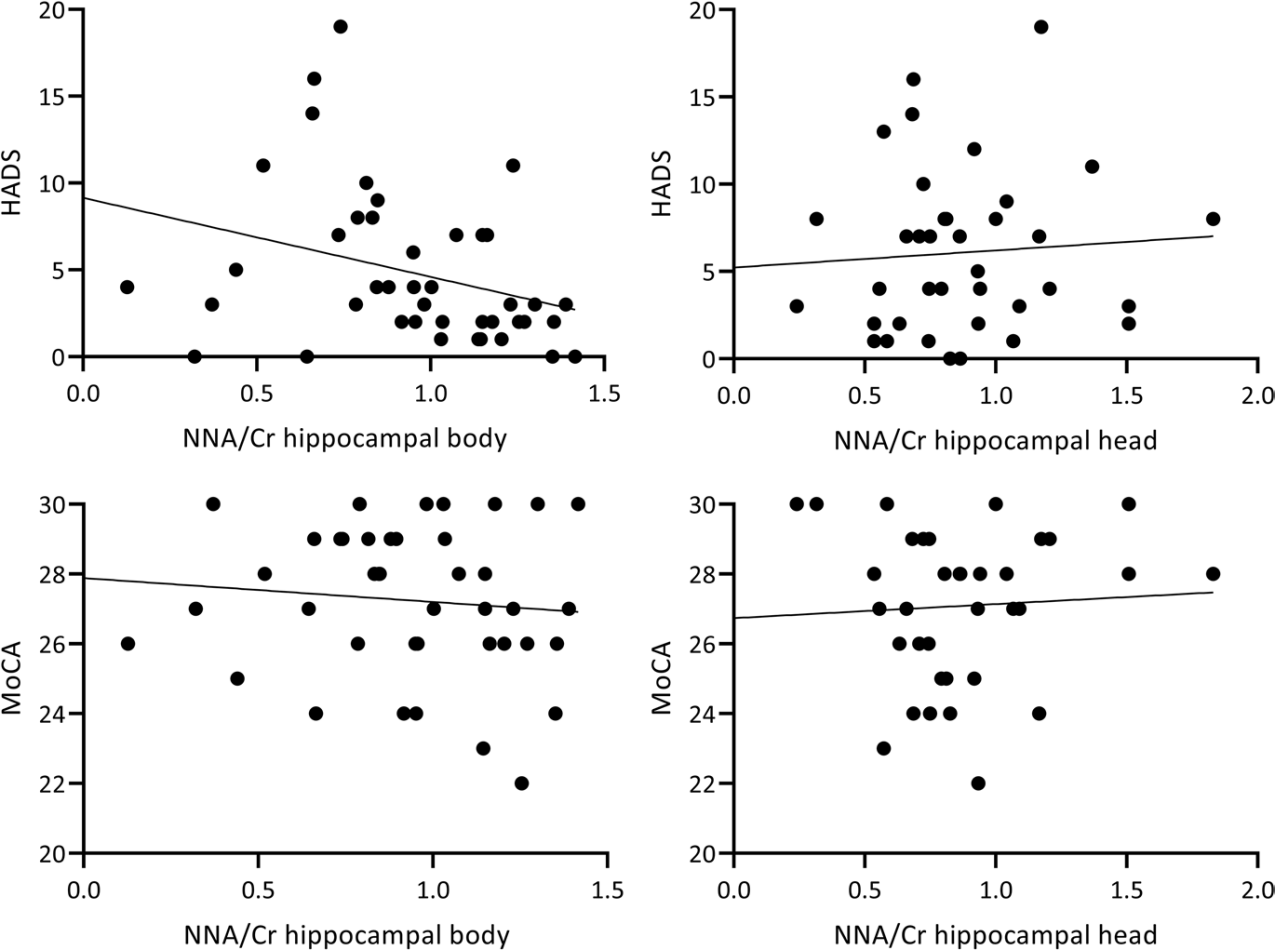
Scatter plot of rawHADS and MoCA scores versus raw NAA/Cr level in the hippocampal body and head

A significant negative association was present between HADS score and NAA/Cr in the hippocampal body but not hippocampal head, with lower NAA/Cr associated with poorer psychological health (Fig. 3, Table 6). There was no significant effect of age, sex, education, or hippocampal volume on HADS score. No relationships between HADS scores and Cho/Cr were uncovered (Table 6).

**Table 6:** General linear model results with MoCA and HADS scores as outcomes and NAA/Cr in the hippocampal body and head, or Cho/Cr levels in the hippocampal body and head as predictors

	NNA/Cr						Cho/Cr					
	Body			Head			Body			Head		
	B	CI	*p*	B	CI	*p*	B	CI	*p*	B	CI	*p*
MoCA	− 4.74	[− 8.47– − 1.02]	**0.02**	− 0.35	[− 3.90–3.20]	0.83	− 1.31	[− 5.03–2.42]	0.46	6.48	[− 7.28–20.23]	0.33
HADS	− 12.16	[− 21.00– − 3.31]	**0.01**	6.06	[− 1.54–13.65]	0.11	− 5.00	[− 13.88–3.89]	0.25	11.38	[− 11.46–34.23]	0.31

General linear models with MoCA or HADS as outcomes variable across all participants (SEG&CG). NAA/Cr or Cho/Cr from MRSI voxels in the hippocampal head and body were included as predictors in the same model corrected for sex, age, education, and right hippocampal volume. *HADS*, Hospital Anxiety and Depression Scale; *MoCA*, Montréal Cognitive Assessment Scale. *p*-values < 0.05 are in bold font

Performance on MoCA was associated with NAA/Cr in the hippocampal body but not in the head, with a lower level of NAA/Cr linked to slightly higher scores, correcting for age, sex, education, and hippocampal volume in the same model (Fig. 3, Table 6). There was no significant effect of age, sex, education, or hippocampal volume on MoCA score. No relationships between MoCA scores and Cho/Cr were uncovered (Table 6).

## Discussion

This is the first exercise RCT in older adults examining metabolites in the hippocampus, a region considered highly modifiable by training and physical activity and a key structure in dementia. Opposed to our predictions, the CG had a higher NAA/Cr in the hippocampal body than the SEG after 3 years of intervention. Likewise, neither change in VO_2peak_, increasing VO_2peak_ from baseline to 3-year intervention, nor VO_2peak_ at 3 years were associated with more favorable hippocampal metabolites, as predicted based on the cardiorespiratory fitness hypothesis [38]. Furthermore, greater exercise intensity, which is connected to larger gains in cardiorespiratory fitness [63], was negatively associated with Cho/Cr in the hippocampal body.

The CG and SEG were well-matched at baseline, and both trained according to their specification with the SEG exercising at a higher intensity as intended. Still, the CG had a significantly higher level of NAA/Cr in the hippocampal body compared to the SEG. NAA/Cr is considered a marker of neuronal viability as it is located to neuronal mitochondria which are enriched in the synapses [64]. The lower NAA/Cr in the SEG suggests a reduction in synaptic health in the SEG even after controlling for hippocampal volume. We have previously shown that hippocampal atrophy rate from baseline was greater in the right hippocampus (where MRSI data were acquired) in the SEG compared to the CG after 3 and 5 years of intervention [20]. Our results were unexpected given the prevailing view of exercising being particularly beneficial for hippocampal structure and function. However, the literature is inconsistent with two recent meta-analyses reporting no effect of exercising on hippocampal volume [65, 66], while another finding reports a small positive effect on right and left hippocampus in older adults [67] suggesting that there are unanswered questions related to exercising and the hippocampus in humans. The lower level of NAA/Cr in the hippocampal body of SEG in our study is in line with the greater rate of atrophy located specifically to the hippocampal body in this group after 3 years [20]. Since we corrected for hippocampal body volume, the lower NAA/Cr level implies that the effect of SEG membership on hippocampal metabolites surpassed that explained by hippocampal volume reduction, suggesting that structural MRI and MRSI provide additive information. The group difference in NAA/Cr in the hippocampal body of CG and SEG was observed despite similar VO_2peak_ levels in these groups. This concurs with our finding that none of the VO_2peak_ measures were related to hippocampal metabolite levels. Interestingly, the previous observational exercise study implementing single-voxel MRS found a higher NAA/Cr ratio in the frontal cortex in the endurance-trained group than their sedentary counterparts [34]. The endurance-trained group had a similar physical activity level as our CG and age-average VO_2max_ levels, like our participants [68]. This suggests that the levels of physical activity/exercise in our CG (and Gonzales et al.’s endurance training group) combined with an age-average VO_2max/peak_ level provide brain health benefits. Contrary to the findings of Gonzales et al., however, we did not uncover a group difference in Cho/Cr, although a trend was evident (CG > SEG), but the effect sizes were small and larger samples are needed to determine group differences in Cho/Cr than NAA/Cr based on our data. Nevertheless, the negative relationship between Cho/Cr and exercise intensity across all participants (SEG&CG) implies that hard cardiorespiratory training had a negative effect on the density of cellular membranes across all neural cells, not just affecting neurons in the hippocampus. Overall, our findings, together with those of Gonzales et al., indicate that there might be a reversed *J* or inverted *U*-shaped relationship between training intensity and brain health outcomes. This concurs with previous descriptions of a reversed *J*-shaped relationship between exercise and premature mortality/cardiovascular disease, where a very high dose of strenuous activity is associated with higher risks compared to less extreme doses while being sedentary comes out worst [69]. Our findings imply that both neurons and glial cells in the hippocampus are sensitive to exercising and that following national physical activity guidelines, having an age-average VO_2peak_ level [68], and not performing training at very high-intensity levels might be ideal for preserving hippocampal metabolites for older adults.

There was no relationship between change in VO_2peak_ from baseline to 3 years, being in the group with an increasing versus declining VO_2peak_ from baseline to 3 years of intervention, or VO_2peak_ at 3 years and hippocampal metabolite levels. Thus, no support for the cardiorespiratory fitness hypothesis was found. This could be due the inclusion of participants from the general population with average VO_2peak_ values [68]. Additionally, both the SEG and CG were physically active. Many studies include sedentary participants allocated to exercising versus remaining sedentary [14, 18, 34]. In our study, however, the CG diligently followed the national guidelines for physical activity, with 87.1% adherence rate. Although the CG were physically active, they exercised with a lower intensity level compared to SEG as shown by lower scores on the Borg scale and a lower percentage of participants following the HIIT protocol. The only previous MRS study on exercise effects reported a significant association between VO_2max_ and NAA/Cr in the frontal cortex [34]. We did not find a similar association in the sensitivity analysis limited to participants who reached VO_2max_ (*n* = 37). The discrepancy could arise from the broader distribution of VO_2max_ in the Gonzales et al. study due to their inclusion of a sedentary control group. Their VO_2max_ values spanned 34 units, while our VO_2peak_ spanned 16 units based on the SDs. A greater range of VO_2max/peak_ values is present when sedentary and exercising individuals are combined in the same study and a greater percentage gain in VO_2max/peak_ is found in sedentary people who begin to exercise [70]; likewise, age may play a role, resulting in discrepant results. The relatively fit participants in the SEG and CG remained remarkably stable in measures considered to be positively affected by exercising such as BMI, blood glucose, and mood from baseline to 3 years [71]. Given the well-matched groups, stable clinical health in both the SEG and CG, lack of association between VO_2peak_ and hippocampal metabolites, and the lower NAA/Cr level in the hippocampal body in the SEG most likely stemmed from the intervention. Indeed, NAA/Cr and Cho/Cr ratios were lower in both voxels in the SEG compared to the CG group, albeit they did not reach statistical significance, pointing to a general trend of reduced levels of neurochemicals.

All significant findings related to effects of exercising on hippocampal metabolites were uncovered in the hippocampal body only, but not its head. The hippocampal head volume has previously been suggested as linked to higher levels of cardiorespiratory fitness [14]. In this cohort though, the hippocampal body was the region with greater atrophy rate in the SEG compared to CG [20], providing an independent line of evidence for the hippocampal body as being susceptible to negative effects of certain types of training in older adults. High-intensity training has been shown to affect hippocampal perfusion and increase lactate levels [15, 72–75] and stress responses [76–78] in older adults and animals. Any or all these mechanisms can influence neuronal and glial metabolism and lead to the differences between SEG or CG, and the association related to exercise intensity.

Higher psychological distress, as measured with HADS, was associated with lower NAA/Cr in the hippocampal body across both groups. As can be seen in Fig. 3, there was a flooring effect in the HADS score as many participants did not report mental health problems. There were six participants, three in each group, with HADS scores > 11 which is considered a cutoff for a clinically relevant level of psychological distress [41, 79]. These participants had similarly high scores on HADS at earlier and later assessments, suggesting stable high mental health problems. Since the six individuals with high HADS scores were equally divided between SEG and CG, and mean HADS scores was slightly higher but not significantly, in the CG group, it is unlikely that HADS scores contributed to the observed CG versus SEG group difference in NAA/Cr sin the hippocampal body. A relationship between NAA/Cr and HADS scores were not present in the hippocampal head. This might be due to the observed differences in NAA/Cr levels in the MRSI voxel in the head and body of the hippocampus and their relationship to clinical measures. If/Whether neurochemicals measured in different hippocampal voxels with MRSI are differently associated with clinical measures, needs to be verified in other cohorts. A lower level of NAA/Cr has been reported with single-voxel MRS in the hippocampus of bipolar patients compared to healthy control earlier [80], providing some support for an effect of psychological distress on hippocampal NAA/Cr levels.

The lower NAA/Cr level uncovered in the SEG group could have cognitive implications [81, 82], but we did not detect evidence of this. Firstly, a lower NAA/Cr level in the hippocampal body was negatively associated with general cognitive ability (MoCA scores) across both groups, i.e., lower level and better MoCA scores, although the relationship was weak. Secondly, no difference were detected in MoCA scores between SEG versus CG in the MRS cohort (Table 2) or the entire RCT Generation 100 study SEG and CG groups after 5 years of intervention [83]. MoCA is a screening tool for mild cognitive impairment [45], and has a limited resolution with a notable ceiling effect, as can be seen in Fig. 3. This is a shortcoming of this measurement and could affect the results. We used the raw scores of the test, and corrected for age, sex and level of education in the statistical model, which are variables known to be associated with performance on the MoCA test [84, 85]. Only body NAA/Cr, not age, sex, education, right hippocampal volume, nor head NAA/Cr, was associated with MoCA performance in this sample. Even though significant, the variance in MoCA scores explained by NAA/Cr in the hippocampal body was much lower than HADS scores explained. To further complicate matters, the relationship between NAA/Cr in the hippocampal head and MoCA scores was not significant, but very weakly positively correlated as evidenced in both the statistical analysis and the scatter plot. A strong positive relationship between NAA/Cr in gray matter regions of interests using single-voxel MRS has been reported previously [82, 86]. One could speculate that the differences in the NAA/Cr in the hippocampal head and body demonstrated in the within-subject analysis could be related to the functional specialization along the hippocampal long axis [87]. This could potentially explain our findings, but it is highly speculative and needs validation.

Medial temporal lobe NAA/Cr has been suggested as a potential marker of dementia [88, 89]. Two early studies using single-voxel MRS reported an uncorrected group difference in NAA/Cr levels in the right hippocampus of 16.5% between controls and dementia patients [29], and the left hippocampus of 17.9% between controls and mild cognitive impairment (MCI) patients [30]. The uncorrected mean percentage difference between NAA/Cr in SEG and CG was 17.9% in our study. Still, we did not observe a negative effect of NAA/Cr level on general cognition, but rather a weak positive effect. Other MRS studies in the precuneus of patients with MCI or dementia consistently report that lower NAA/Cr is associated with or predicts lower general cognitive function, MCI, or dementia [29, 90–92]. This could imply that the observed difference in NAA/Cr in the hippocampal body is still within what is normal or supports normal functioning. The group difference in NAA/Cr might thus be within the range of typical aging but could render the SEG participants with less brain reserve as they age. Finally, none of the covariates (sex, age, education, and hippocampal volume) was associated with MoCA or HADS scores, implying that MRSI might be more sensitive to functional outcomes than the standard variables described as linked to mental health and cognitive outcomes.

## Strengths and Limitations

The main strength was the study design with recruitment from the general population into an RCT, the detailed clinical assessment from baseline, objective VO_2peak_ measurements, and long-term supervised exercising for three years. Participants adhered well to their assigned training regime, but since also the CG subjects were meticulous in their training, there were few differences between the SEG and CG, except for exercise intensity. In Norway, 32% of older adults (65 + years) follow the national physical activity guidelines [93], while in the present study, 87.1% in the CG did, demonstrating remarkable compliance. The excellent compliance to the national physical activity guidelines makes it difficult to generalize the results from CG to the general population.

All statistical models were corrected for variables known to be connected to hippocampal size such as age [94], sex [95], and education [96] as well as volume per se. Many statistical analyses were performed in this study following the main outcome analysis of the intervention group effect (SEG versus CG). We did not employ methods for correcting for multiple comparisons. Implementation of Bonferroni correction in the across all participant general linear model results related to exercise characteristics would give an adjusted *p*-values < 0.005 (*p* = 0.05 divided by 10 analyses for NAA/Cr and 10 analyses for Cho/Cr), and for the prediction of MoCA and HADS score, *p*-values < 0.025 (*p* = 0.05 divided by two analyses). Thus, the *p*-values uncovered would have been significant also after implementation of such correction.

A limitation of the study was the low number of participants in whom we were able to obtain acceptable MRSI data. Still, the final number was similar to the *n* in the only previous study investigating the effect of exercise on brain metabolites [34]. Nevertheless, in this age group, single-voxel MRS could be a good alternative to MRSI due to better SNR and shorter acquisition times. Still, using MRSI with sLASER for investigating hippocampal metabolites in distinct long-axis regions of the hippocampus is novel and provided a map of the spatial variation of neurochemicals not obtained with single-voxel MRS. The fact that we found lower NAA/Cr in the hippocampal body, the same region where we have previously shown greater atrophy rate in the SEG compared to the CG [20], and that there were significant differences in NAA/CR levels between the hippocampal head and body within subjects supports the notion that MRSI with sLASER reflects variability between anatomical regions even within a structure as small as the hippocampus. sLASER has the advantage to provide a sharper voxel localization and low sensitivity to B0 and B1 inhomogeneities. These features could be advantageous for MRSI of the hippocampus considering its small size and proximity to structures such as the petrous bone and sinuses which can cause broader linewidth and relatively low spectral resolution compared to other brain regions. Most studies that have applied MRS to study neurochemicals in the hippocampus have implemented large voxels compared to the size of the hippocampus and higher magnetic field than 3T [97–101]. However, sLASER suffers from longer minimum TE and higher RF energy deposition being directly limited by specific absorption rate restrictions which must be considered, especially at higher magnetic fields. The Cr resonance is widely used as internal reference as its level is high and comparable in different tissue types in the brain. Since Cr is present in neural and glial cells, the interpretation of Cr levels can be difficult, and any difference in a ratio can arise from differences in either the numerator (i.e., NAA or Cho) or denominator (i.e., Cr). Moreover, other metabolites of interest could have been examined (e.g., myoinositol/Cr levels) from the existing MRSI data, which have been shown to be related specifically to MCI and dementia [89, 91, 92, 102, 103]. In the future, more advanced MRS methods with J-coupling could be employed to investigate other neurochemicals of importance, such as excitatory (glutamate) and inhibitory (γ-aminobutyric acid) neurotransmitters, as well as energy metabolites like lactate or β-hydroxybutyrate [104]. Also, unlike the structural MRI protocol, MRSI was only collected after 3 years, the original RCT endpoint. The problems experienced during the MRSI acquisition at 3 years, leading to loss of 22% of the MRSI data due to motion, small hippocampal volumes, and technical processing/analysis issues, led us to decide not to repeat MRS acquisition at 5 years, the updated RCT endpoint. As a comparison, we did not lose any structural MRI data due to motion.

The lack of longitudinal MRSI data is a shortcoming of this study. It is possible differences existed in hippocampal metabolites between groups already at baseline, but given the lack of SEG and CG differences on other variables, this is likely not to be a serious problem. The results at the 3-year timepoint would have been even stronger if repeated at 5 years.

## Conclusion

This study demonstrated that SEG had significantly lower NAA/Cr in the hippocampal body after 3 years of supervised exercise intervention compared to an active CG following the national physical activity guidelines. Moreover, higher exercise intensity, irrespective of group, was associated with lower Cho/Cr in the same part of the hippocampus. The MRSI results are in line with structural MRI results from the same cohort showing that the SEG group had higher rates of hippocampal atrophy [20] and white matter hyperintensity growth [105] than the CG. Additionally, our results demonstrate that sLASER allows for a detailed investigation of spatially specific metabolic profiles along the hippocampal long axis, and that the neurochemical profiles were more closely associated with functional outcome than for instance the hippocampal volume. In summary, this study demonstrated that diligently following the national physical activity guidelines and training at moderate intensity were linked to the healthiest neurochemical profile in the hippocampus and that metabolites from both neurons and glia cells were sensitive to exercise regimes.

## Data Availability

Data used in this manuscript can be accessed by contacting the corresponding author. Access to data by qualified investigators will be subject to ethical and scientific review (to ensure the data is being requested for valid scientific research) and must comply with the European Union General Data Protection Regulations (GDPR), Norwegian laws and regulations, and NTNU regulations. The completion of a material transfer agreement (MTA) signed by an institutional official will be required.
